# Reciprocal regulation of TWIST1 and OGT determines the decitabine efficacy in MDS/AML

**DOI:** 10.1186/s12964-023-01278-y

**Published:** 2023-09-22

**Authors:** Hongjiao Li, Yi Wang, Shuang Feng, Kaijing Chang, Xinwen Yu, Fenfang Yang, Haozhe Huang, Yuanbo Wang, Xiang Li, Feng Guan

**Affiliations:** 1 Key Laboratory of Resource Biology and Biotechnology Western China, Ministry of Education; Provincial Key Laboratory of Biotechnology, College of Life Sciences, Northwest University, Xi’an, 710069 China; 2 Department of Hematology, Provincial People’s Hospital, Xi’an, 710068 China; 3 Institute of Hematology, School of Medicine, Northwest University, Xi’an, 710069 China; 4 College of Life Science, Northwest University, 229 Taibai North Road, Xi’an, 710069 Shaanxi China

**Keywords:** TWIST1, OGT, O-GlcNAc, DAC resistance, MDS/AML

## Abstract

Chemoresistance poses a significant impediment to effective treatment strategies for myelodysplastic syndromes (MDS) and acute myeloid leukemia (AML). Our previous study unveiled that oncogene TWIST1 interacted with DNA methyltransferase 3a (DNMT3a) to regulate the decitabine (DAC) resistance in MDS/AML. However, the underlying mechanism of TWIST1 dysregulation in DAC resistance remained enigmatic. Here, we found that O-GlcNAc modification was upregulated in CD34^+^ cells from MDS/AML patients who do not respond to DAC treatment. Functional study revealed that O-GlcNAcylation could stabilize TWIST1 by impeding its interaction with ubiquitin E3 ligase CBLC. In addition, as one typical transcription factor, TWIST1 could bind to the promoter of O-GlcNAc transferase (OGT) gene and activate its transcription. Collectively, we highlighted the crucial role of the O-GlcNAcylated TWIST1 in the chemoresistance capacity of MDS/AML clonal cells, which may pave the way for the development of a new therapeutic strategy targeting O-GlcNAcylated proteins and reducing the ratio of MDS/AML relapse.

Video Abstract

**Supplementary Information:**

The online version contains supplementary material available at 10.1186/s12964-023-01278-y.

## Background

Myelodysplastic syndromes (MDS) are clonal disorders of hematopoietic stem/precursor cells characterized by ineffective hematopoiesis, generally presenting as single or multilineage cytopenia in peripheral blood [1]. ~ 30% of MDS patients are at risk of transforming to secondary acute myeloid leukemia (AML) [2]. Previously, we demonstrated that CD34^+^ marrow cells in advanced MDS express high levels of TWIST1 [3]. As a transcription factor (TF), TWIST1 plays a pivotal role in self-renewal and differentiation of mesenchymal stem cells and hematopoietic stem cell maintenance by binding to E-box motif of target genes and regulating their transcription [4, 5]. In the follow-up study, we demonstrated that TWIST1 mediates decitabine (DAC) resistance of MDS/AML clone cells by binding to the gene promoter of DNA methyltransferase 3 (DNMT3) [6]. These data illustrated the vital role of TWIST1 in the pathophysiology of hematopoietic malignancies. However, the upstream events that lead to the dysregulated expression of TWIST1 remain unclear.

In many cases, posttranslational modification (PTMs) that has been shown to regulate the expression of TFs. Among different types of PTMs, O-linked β-N-acetylglucosamine (O-GlcNAc) modification is a dynamic and reversible event catalyzed coordinately by O-GlcNAc transferase (OGT) or glycoside hydrolase O-GlcNAcase (OGA), which are responsible for the addition or removal of GlcNAc via an O-linkage to serine (Ser) or threonine (Thr) residues of nuclear, cytoplasmic, mitochondrial and other proteins, respectively [7].

As one molecular barcode, O-GlcNAcylation is involved in regulation of a variety of cellular processes. O-GlcNAcylated proteins mediate direct cell–cell interactions and control numerous cell fate specifications. It is well known that many TFs could be O-GlcNAcylated, and O-GlcNAcylation can affect their translocation, DNA binding capacity and stability [8, 9]. For example, O-GlcNAcylation protects TF Sp1 from proteasomal degradation and promotes its nuclear localization in breast cancer [10]. O-GlcNAcylation reduces of the stability of Forkhead Box A1, leading to the downregulation of the pro-apoptotic protein Bim, thereby inhibiting cell apoptosis [11]. Our 2022 study found that O-GlcNAcylation at T717 on STAT3 facilitates epithelial-to-mesenchymal transition process by promoting STAT3 phosphorylation [12]. To date, the existence of O-GlcNAcylation on TWIST1 and its role in TWIST1 mediated DAC resistance in MDS/AML were not explored.

In this study, we found the elevated OGT and O-GlcNAc level in MDS/AML patients who did not respond to DAC treatment. Mechanism study further explored how O-GlcNAc modification influenced the TWIST1 stability and TWIST1 modulated OGT expression in turn and revealed how reciprocal regulation of TWIST and OGT determined the DAC efficacy in MDS/AML.

## Methods

### Cell lines and cell culture

Human myeloid leukemia cell line KG1a, MDS cell line SKM1 and embryonic kidney cell line HEK-293 T were cultured and propagated as previously described [6]. KG1a, SKM1, TWIST1 overexpressed KG1a (KG1a-TWIST1), TWIST1 silenced SKM1 (SKM1-shTWIST1) cells, and decitabine-resistant KG1a (KG1a-DAC-R) cells were established and cultured as previously described [6].

The mononuclear cells were isolated from bone marrow aspirates of MDS/AML patients (Table S1) using Ficoll-Hypaque gradient centrifugation as previously described [13]. CD34^+^ cells were sorted using the CD34 microbeads Kit (#130-046-702, Miltenyi Biotechnology company; Bergisch Gladbach, Germany) [14]. In accordance with the Declaration of Helsinki, written informed consent were obtained from all patients and healthy donors (HD).

### Quantitative real-time polymerase chain reaction (qRT-PCR)

Total RNA was isolated using RNA pure Tissue & Cell Kit (#CW0560, Cwbiotech; Beijing, China), and cDNA was synthesized using ReverTra Ace qPCR RT Kit (#FSQ-101, Toyobo; Osaka, Japan), according to the manufacturer’s protocol. Amplification and detection were performed with Power SYBR Green Master Mix (#CW0659S, Cwbiotech) and Gentier 48R System (Tianlong Technology, Xi’an, China) using the listed primers (Table S2). The observed copy numbers of target genes were normalized to internal GAPDH expression and quantified by 2^−ΔΔCt^ method [15, 16].

### Enzyme-linked immunosorbent assay (Elisa)

One hundred µL cell lysates (10 µg/mL) in coating buffer was placed in each well of a microtiter plate and incubated at 4 °C overnight. Coating solution was removed, and the plate was washed three times with 200 μL 0.05% Tween 20 in PBS. The antibody against O-GlcNAc (#ab2739, Abcam, Cambridge, MA, USA) and the secondary antibody conjugated horseradish peroxidase (HRP) was added sequentially. After incubating at 37 °C for 1 h, 100 μL tetramethylbenzidine (TMB; #P0209, Beyotime) was added and the intensity of chromogenic reaction was determined at 490 nm using a plate reader (Bio-Rad Laboratories, Hercules, CA, USA).

### Cell proliferation assay

Cells were stained with EdU Alexa Fluor 647 kit (#KGA334-50, Keygen; Jiangsu, China) according to the manufacturer’s protocol. The stained cells were analyzed by flow cytometry (FACS; ACEA Biosciences; San Diego, CA, USA).

### SDS-PAGE and western blotting

Cells were collected and lysed with RIPA buffer (50 mM Tris, pH 7.2, 1% Triton X-100, 0.5% sodium deoxycholate, 0.1% SDS, 150 mM NaCl, 10 mM MgCl_2_ and 5% glycerol) containing 1% protease inhibitor PMSF. Protein concentration was determined using BCA Protein Assay Kit (#P0011, Beyotime). Proteins (25 μg) from each lysate were separated by electrophoresis in a 10% polyacrylamide resolving gel and transferred onto a polyvinylidene difluoride (PVDF) membranes. After blocking with 3% BSA (#ST023, Beyotime) in Tris-buffered saline containing 0.1% Tween-20 (TBS-T) at room temperature for 1 h, membranes were incubated at 4 °C overnight in TBS-T containing following antibodies against TWIST1 (#sc-81417, Santa Cruz; CA, USA), Tubulin (#2146 s, Cell Signaling Technology; Beverly, MA, USA), O-GlcNAc (#ab308178, Abcam) and OGT (#ab177941, Abcam) followed by the addition of secondary antibody conjugated with HRP (#A0208, #A0216, Beyotime). Bands were visualized by enhanced chemiluminescence (ECL; Vazyme Biotech; Nanjing, China).

### Co-immunoprecipitation (Co-IP)

The Co-IP assay was performed as described previously [17]. Cell lysates were incubated with antibody against TWIST1, O-GlcNAc or Flag (#14793, Cell Signaling Technology) at 4 °C overnight, and then incubated with *Protein A/G* agarose (#sc-2003, Santa Cruz) at 4 °C for 12 h. Agarose was washed with PBS and collected by centrifugation at 2,000 g for 5 min. Samples were collected by boiling in 1 × SDS loading buffer for 20 min and subjected to western blotting analysis.

### In vitro O-GlcNAcylation

The in vitro O-GlcNAcylation reaction was performed as described previously [18]. Briefly, TWIST1 (in pET28a plasmid) bearing 6 × His tag and OGT (in pEGX-4T1 plasmid) bearing GST tag were separately overexpressed in *Escherichia coli* BL21 (DE3) and purified by commercial Purification Kits (#P2226, Beyotime; Haimen, China). His-tagged TWIST1 (10 μg), GST-tagged OGT (20 μg) and 2.5 mM UDP-GlcNAc (gifted from Prof. Junqiang Fang, Shandong University; China) were added in 100 μL reaction buffer (125 mM NaCl, 1 mM EDTA, and 20 mM Tris–HCl, pH 7.4), and reacted at 37℃ for 6 h. The mixture was further assayed for O-GlcNAcylation site analysis by mass spectrometry (#QE HF-X, ThermoFisher, MA, USA).

### Analysis of O-GlcNAc sites on TWIST1

O-GlcNAc sites on TWIST1 was analyzed by LC–MS [19]. The O-GlcNAcylated TWIST1 in vitro were separated by SDS-PAGE, and gel pieces were dehydrated with acetonitrile (#1.00030, Sigma-Aldrich; MO, USA), reduced with DTT (#D0632, Sigma-Aldrich), alkylated with IAA (#I2273, Sigma-Aldrich) and digested with trypsin (#4370285, Sigma-Aldrich). Digested peptides were dissolved in 0.1% TFA (#T6508, Sigma-Aldrich) and loaded and separated with a nano column of PepMap C18 column with U3000 RSLCnano system (ThermoFisher). Buffer A was 0.1% TFA in water, and buffer B was 0.1% TFA in 80% ACN. Peptides were separated by gradient of 2% buffer B to 8% buffer B for 4 min, 8% buffer B to 38% buffer B for 79 min, 30% buffer B to 100% buffer B for 7 min, holding with 100% buffer B for 4 min. MS data was acquired by data-dependent method with top speed mode on QE HF-X with following parameters: spray voltage 2 kV; S-lens RF level 50; capillary temperature 300 ºC; full scan resolution 60,000 at m/z 200; full scan automatic gain control (AGC) 3e6 with maximum fill time 30 ms; mass range of full mass 350–1500; high-collision dissociation (HCD) scans with resolution 15,000 at m/z 200 and AGC target at 5E4; maximum ion injection time for HCD scans 45 ms; fixed first mass 110; normalized collision energy (CE) 27; precursor ions with single, unassigned charge states removed from fragmentation selection; dynamic exclusion 40 s; cycle time 3 s.

LC–MS data was analyzed by Maxquant (Max Planck Institute of Biochemistry). Database searching was performed with following parameters: TWIST1.fasta as the database, a precursor mass tolerance of 10 ppm, a fragment mass tolerance of 0.02 Da, and fixed modifications of carbamidomethyl on C, oxidation on M, and HexNAc on S/T as dynamic modifications. Fixed Value PSM validator was used for validation. IMP-ptmRS algorithm was used for O-GlcNAc location. Minora Feature Detector was used for quantification. O-GlcNAc sites with a site location probability greater than 0.75 were confidently located.

### Half-life assay for TWIST1 expression

KG1a-TWIST1 and SKM1 were treated with 50 μM cycloheximide (CHX, # HY-12320, MedChemExpress; NJ, USA) to inhibit protein synthesis, and harvested at the indicated time points. The cell lysate lysates were prepared and subjected to SDS-PAGE and western blotting. The relative half-life of TWIST1 was calculated based on its expression at the indicated time points.

### Proximity ligation assay

Proximity ligation assay was performed with the Duolink PLA kit (#Duo94104, ThermoFisher) according to the manufacturer’s instructions. HEK-293 T cells were fixed and incubated overnight with anti-TWIST1 antibody (#ab50518, Abcam; Cambridge, MA, USA) and anti-CBLC antibody (#F-2, Santa Cruz; CA, USA). After multiple washings, cells were incubated successively with PLA probes, ligation solution and amplification solution at 37℃. Cover-slips were mounted, and the images were photographed using Leica TCS SP5 confocal microscope (Leica Microsystems; Mannheim, Germany).

### Chromatin immunoprecipitation assay (ChIP)

The ChIP assay was performed as described previously [20]. Briefly, KG1a-TIWST1 cells were cross-linked with 1% formaldehyde. DNA was sheared to an average size of 200 ~ 500 bp using the sonicator (#Ymnl-1000Y, Immanuel, Nanjing, China). Immunoprecipitation was performed using antibody against TWIST1 (#ab50887, Abcam). Protein A/G agarose was added and rotated for 4 h. The samples were subsequently treated with proteinase K (#ST532, Beyotime) and RNase A (#ST578, Beyotime). Target DNA was extracted using phenol/chloroform and analyzed by PCR with the primers in Table S2.

### DNA pull down assay

DNA probes were amplificated by PCR using primes listed in Table S2. Biotinylated DNA probes (5 μg) modified by EMSA Probe Biotin Labeling Kit (#GS008, Beyotime) were incubated with cell nucleus lysate (500 μg) which extracted by Nuclear Protein Extraction Kit (#R0050, Solarbio; Beijing, China) for 1 h. The mixture was reacted with streptavidin conjugated magnetic beads (#p2151, Beyotime) at 37℃ for 1 h. The magnetic beads were washed by cold PBS for 3 times and collected by magnetic frame. The magnetic beads were collected by boiling in 1 × SDS loading buffer for 20 min and the supernatants were subjected to western blotting analysis.

### Dual luciferase reporter assay

The wild type and mutant regions of OGT promoter were amplified by PCR and cloned into pGL3 basic vector (#E1751, Promega; Madison, WI, USA). HEK-293 T cells were co-transfected with Renilla luciferase expression plasmid (pRL-TK, #D2760, Beyotime) and TWIST1 overexpression vector. After 48 h, luciferase activity was measured with a Dual Luciferase Reporter Assay System (#RG028, Beyotime).

### In vivo mouse model

6–8 week-old B-NSG mice (NOD-Prkdc^scid^*IL2rg*^tm1^/Bcgen, NSG) were irradiated with 180 cGy. A total of 2 × 10^6^ KG1a-TWIST1, KG1a-ko-TWIST1-WT or KG1a-ko-TWIST1-31A) cells were injected into NSG mice through the tail vein. Similarly, MDS cells were transplanted into NSG mice by intravenous injection as previously described [21]. After a 7-day injection period, the mice were categorized into four groups, each consisting of six mice. MDS-transplanted mice were treated with decitabine (DAC, #A3656, 0.2 mg/kg, Sigma-Aldrich or OSMI-1, #HY-119738, MedChemExpress, 0.5 mg/kg) by intraperitoneal injection 2 times per week. Peripheral blood was collected after injecting DAC for 3 times. Mononuclear cells from peripheral blood were stained with antibody against human CD45 (#561865, BD Biosciences; Franklin Lakes, NJ, USA) and analyzed by FACS. Spleen and bone marrow were collected after injecting DAC for 6 times. The cells in spleen and bone marrow were stained with antibody against human CD45 and analyzed by Immunohistochemistry.

### Statistical analysis

Prism 8.0 Statistical Software program (GraphPad Software; La Jolla, CA, USA) was used for statistical analysis. Intergroup means were compared using Student’s t-test, and multiple group comparisons were evaluated by ANOVA with Bonferroni’s post hoc test. Differences at *p* < 0.05 were considered statistically significant. Each experiment was performed at least thrice. Data are presented as mean ± SD.

## Results

### O-GlcNAcylation associated with DAC resistance in MDS/AML

**Fig. 1:**
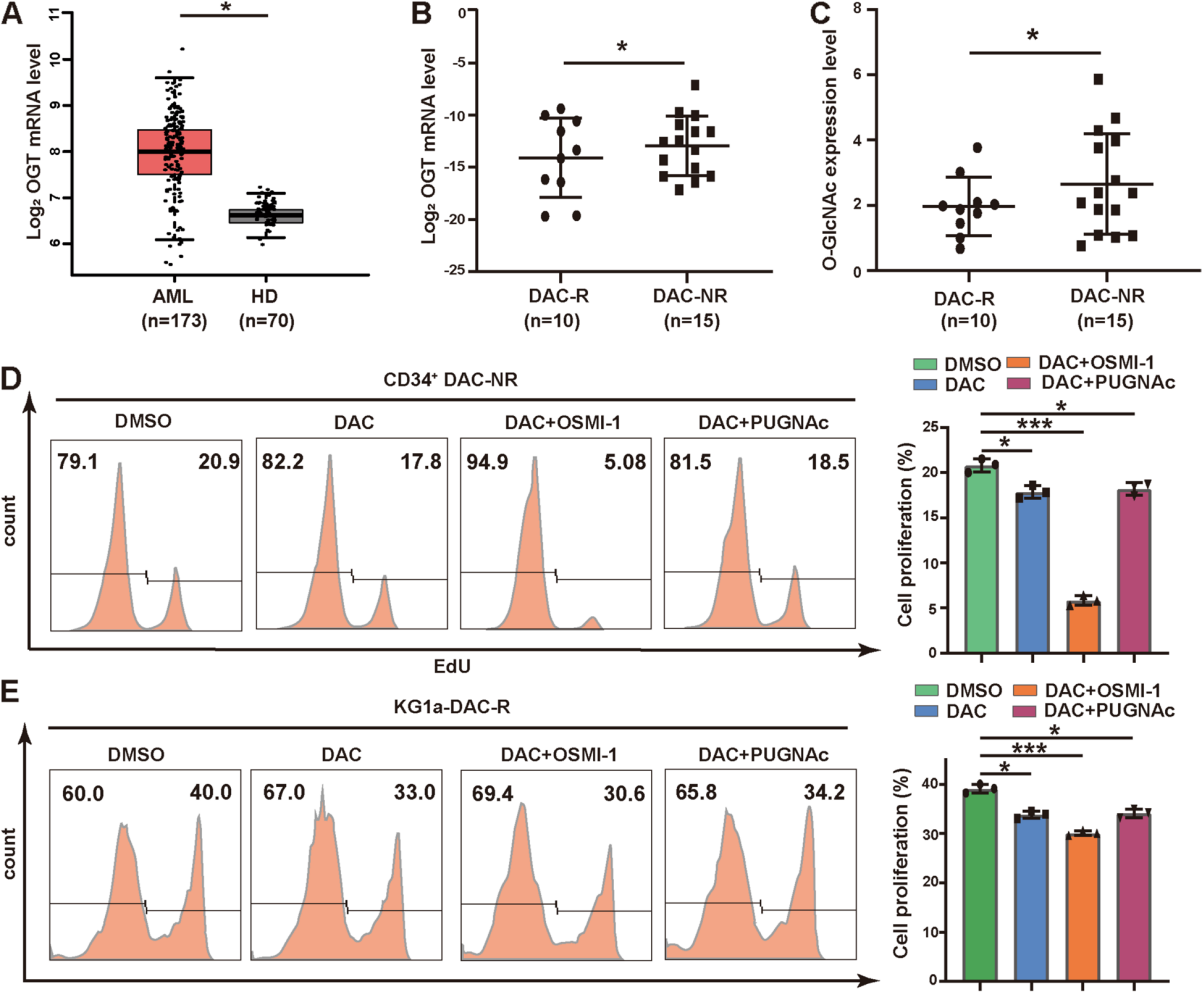
O-GlcNAcylation associated with DAC resistance in MDS/AML. **A** mRNA level of TWIST1 in AML patients *vs* HD in TCGA database. **B**, **C** qRT-PCR analysis of OGT mRNA (**B**) and Elisa analysis of O-GlcNAc level (**C**) in CD34^+^ cells of DAC-R and DAC-NR. **D** Cell proliferation assay. CD34^+^ cells were derived from MDS patients who are non-responsive to DAC and treated with DAC (20 ng/mL) and OSMI-1 (10 μg/mL) or PUGNAc (100 μM) for 48 h. **E** Cell proliferation assay. KG1a-DAC-R cells were treated with DAC (20 ng/mL) and OSMI-1 (10 μg/mL) or PUGNAc (100 μM) for 48 h. *p < 0.05, ***p < 0.001

With the Cancer Genome Atlas (TCGA) database analysis, we found that OGT expression was significantly increased in patients with AML compared to healthy donors (HD) (Fig. 1A). In our collected clinical samples, increased OGT expression and higher O-GlcNAcylation level were presented in CD34^+^ cells from MDS/AML patients who were non-responsive than those who were responsive to DAC treatment (DAC-NR vs DAC-R, Fig. 1B, C).

Consistently, OGT expression and O-GlcNAcylation level of DAC-resistant KG1a cells (KG1a-DAC-R) were significantly increased, compared to its parental KG1a cells (Fig. S1A). The upregulation of OGT and O-GlcNAcylation in DAC treated KG1a and SKM1 cells occurred in time and dose dependent manners (Fig. S1B-E).

The proliferation of hematopoietic stem/progenitor cells (HSC/HPC, CD34^+^) from bone marrow of DAC-NR patients were significantly inhibited by DAC and OGT inhibitor OSMI-1, but not by DAC and OGA inhibitor PUGNAc (Fig. 1D). KG1a-DAC-R showed the similar proliferation as CD34^+^ cells under DAC and OSMI-1 or PUGNAc treatment (Fig. 1E). However, the cell apoptosis of KG1a-DAC-R and CD34^+^ cells did not show significantly change under DAC and OSMI-1 or PUGNAc treatment (Fig. S1F&G). Above data indicate that elevated O-GlcNAcylation may participate in the cellular response to DAC in MDS/AML.

### O-GlcNAcylation stabilizes TWIST1 expression

**Fig. 2:**
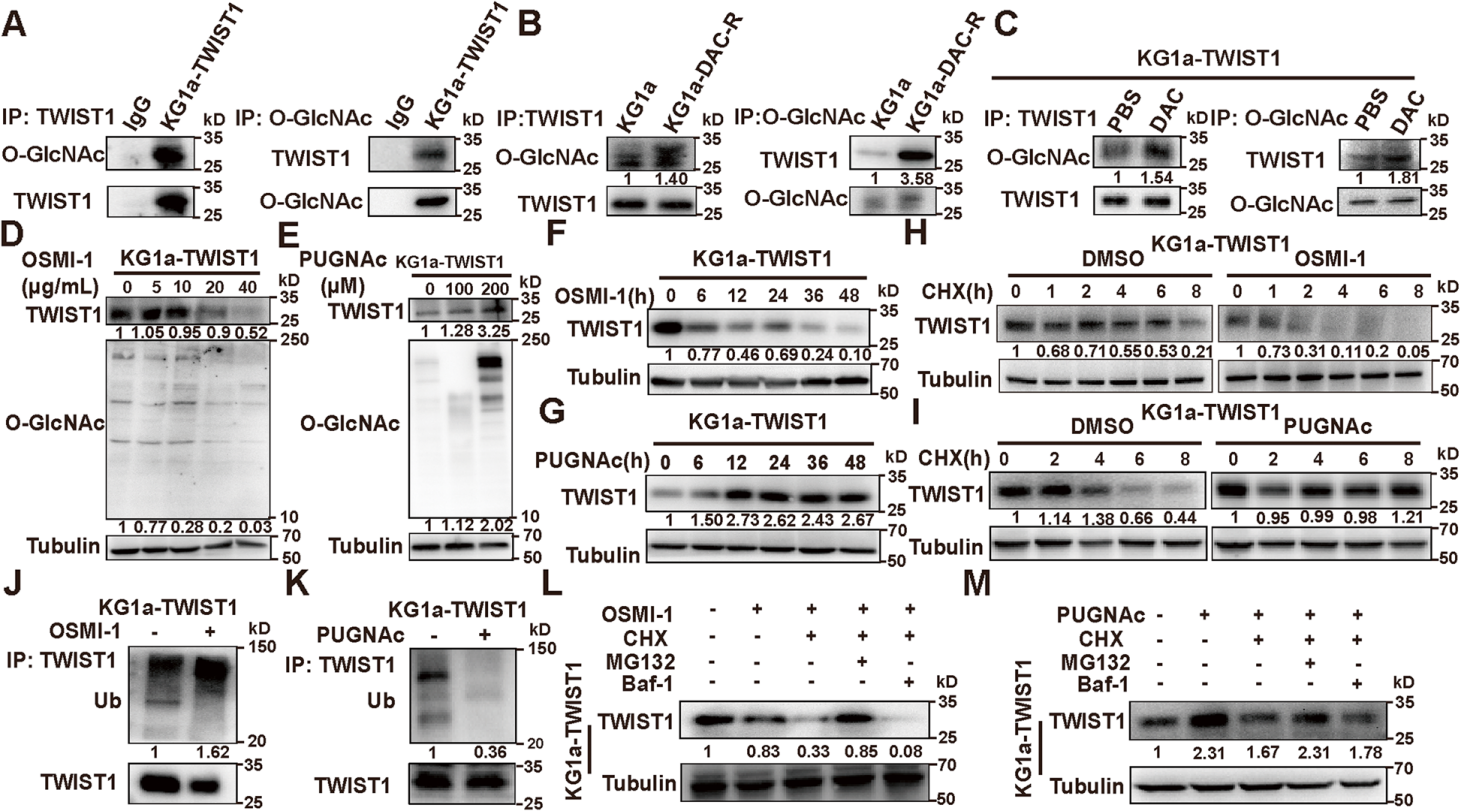
O-GlcNAcylation mediates DAC resistance by stabilizing TWIST1. **A** O-GlcNAc modification of TWIST1 detected by Co-IP and western blotting. Normal mouse IgG was used as negative control. **B** O-GlcNAc modification on TWIST1 in KG1a-DAC-R cells. **C** O-GlcNAc modification of TWIST1 in KG1a-TWIST1 cells after 20 ng/mL DAC treatment for 96 h. **D**, **E** TWIST1 expression in KG1a-TWIST1 cells treated with different concentrations of OSMI-1 (**D**) or PUGNAc (**E**) for 48 h. **F**, **G** TWIST1 level in KG1a-TWIST1 cells treated with 10 μg/mL OSMI-1 (**F**) or 100 μM PUGNAc (**G**) at indicated times. **H**, **I** TWIST1 level in KG1a-TWIST1 after 10 μg/mL OSMI-1(**H**) or 100 μM PUGNAc (**I**) treatment for 48 h and 1 μM CHX treatment for indicated times. **J**, **K** Ubiquitination of TWIST1 in KG1a-TWIST1 cells under 10 μg/mL OSMI-1 (**J**) or 100 μM PUGNAc (**K**) treatment. **L** TWIST1 level in KG1a-TWIST1 cells treated with 10 μg/mL OSMI-1, 1 μM CHX and 5 μM MG132 or 0.5 μM Baf-1 for 48 h. **M** TWIST1 level in KG1a-TWIST1 cells treated with 100 μM PUGNAc, 1 μM CHX and 5 μM MG132 or 0.5 μM Baf-1 for 48 h

In previous study, we demonstrated that increased TWIST1 can interact with DNMT3a, thereby promote the resistance of MDS/AML cells to DAC [6]. As O-GlcNAc can modify many proteins in the nucleus and cytoplasm [19], we speculated the dysregulated TWIST1 was associated to enhanced O-GlcNAcylation level in MDS/AML. Our co-IP data demonstrated the existence of O-GlcNAcylation on TWIST1 in KG1a-TWIST1 and in KG1a-DAC-R cells (Fig. 2A, B, Fig. S2A). Elevated O-GlcNAcylation on TWIST1 was also observed in KG1a-DAC-R and in DAC treated KG1a-TWIST1 (Fig. 2B, C).

With OSMI-1 treatment, O-GlcNAc level and TWIST1 expression were both decreased (Fig. 2D). In contrast, with PUGNAc treatment, O-GlcNAc level and TWIST1 expression were clearly enhanced (Fig. 2E). With the increased timing of OSMI-1 or PUGNAc treatment, TWIST1 expression was significantly decreased or increased (Fig. 2F, G). Similar results were also found in another MDS cell line SKM1 (Fig. S2B-E). Treatment with OSMI-1 or PUGNAc did not show any significant influence on mRNA level of TWIST1 (Fig. S2F-G), indicating that O-GlcNAcylation affects TWIST1 expression at post-translational level.

When treated with CHX, the protein synthesis inhibitor, TWIST1 degradation was accelerated under hypo O-GlcNAcylation condition (Fig. 2H, S2H), but decelerated under hyper O-GlcNAcylation condition (Fig. 2I, S2I). Compared to that in non-treated cells, more ubiquitinated TWIST1 was observed in OSMI-1 treated KG1a-TWIST1 and SKM1 cells (Fig. 2J, S2J), while less ubiquitinated TWIST1 was presented in PUGNAc treated cells (Fig. 2K, S2K). Moreover, proteasome inhibitor MG132, but not lysosome inhibitor Baf-1, could restore the TWIST1 level (Fig. 2L, M, S2L-M). Taken together, O-GlcNAcylation enhances TWIST1 stability through inhibiting proteasomal degradation.

### O-GlcNAcylation at Ser 31 affects TWIST1 stability

**Fig. 3:**
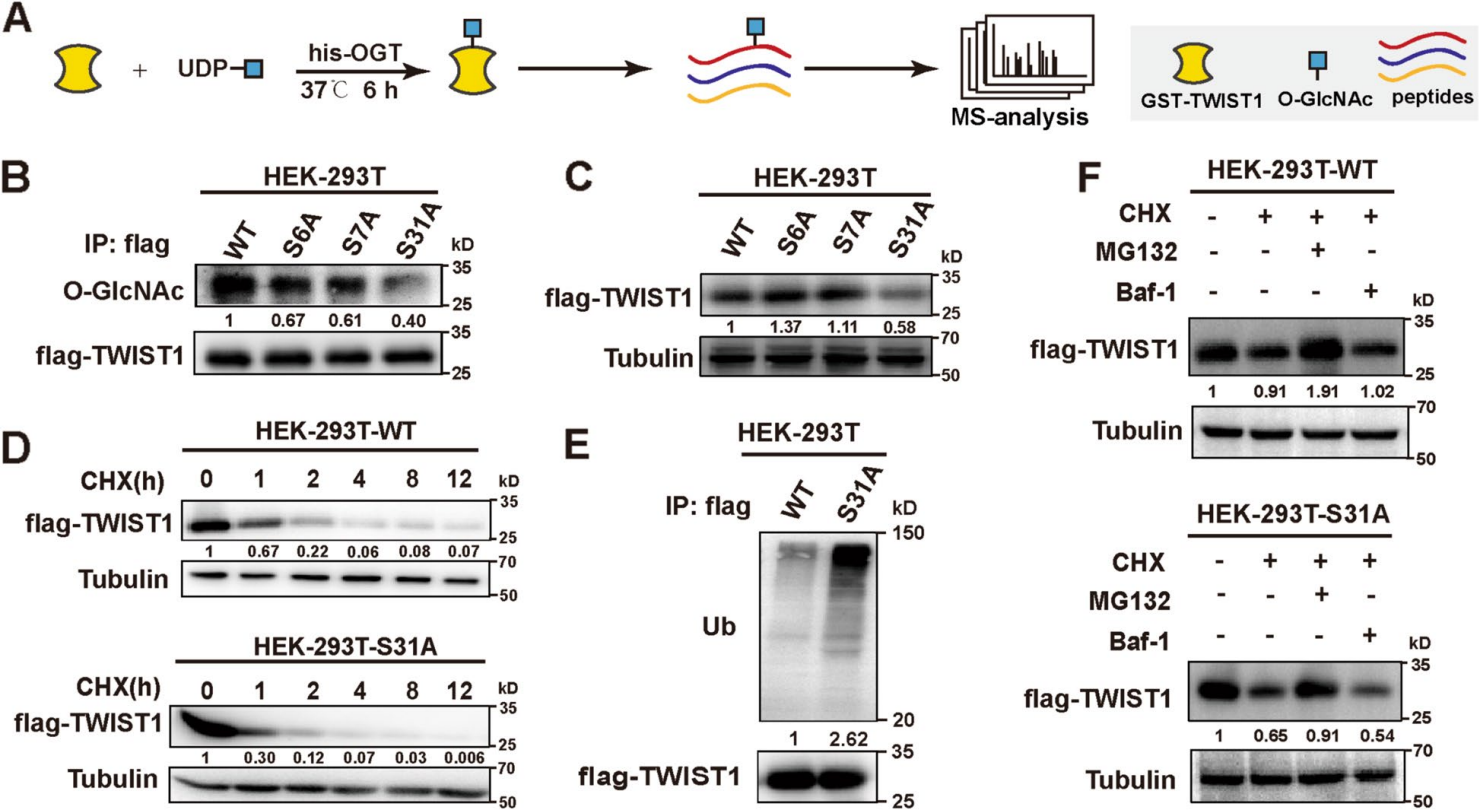
Effect of O-GlcNAcylation at Ser 31 on TWIST1 stability. **A** O-GlcNAclylated GST-TWIST1 in vitro. **B** O-GlcNAcylation level on WT and site-mutated TWIST1 detected by IP/Western blotting. **C** TWIST1 level in HEK-293 T cells transfected with WT or mutation TWIST1. **D** TWIST1 level in HEK293T-WT or HEK-293 T-S31A after 1 μM CHX treatment for indicated times. **E** Ubiquitination of TWIST1 in HEK293T-WT or HEK-293 T-S31A cells. **F** TWIST1 level in HEK293T-WT or HEK-293 T-S31A cells after 5 μM MG132 or 0.5 μM Baf-1 treatment for 8 h

Mass spectrometry results showed that Ser 6, Ser 7 and Ser 31 of TWIST1 were modified by O-GlcNAcylation (Fig. 3A). We hereby overexpressed the individual site mutants (S6A, S7A, S31A) and wild type (WT) of flag-TWIST1 in HEK-293 T cells. Mutations of S31A, but not S6A and S7A, presented the reduced O-GlcNAc levels on TWIST1 (Fig. 3B). With lower O-GlcNAcylation levels, expression of TWIST1 mutation at S31A was reduced (Fig. 3C). CHX chase analysis revealed that TWIST1 stability was impaired by mutation at S31A (Fig. 3D). Meanwhile, TWIST1 mutation at S31A was more ubiquitinated than WT TWIST1 (Fig. 3E). The expression of exogenous mutant TWIST1 at S31A was much lower than that of WT, and this phenomenon was blocked by MG132 (Fig. 3F).

### O-GlcNAcylation on TWIST1 inhibited its binding to CBLC

**Fig. 4:**
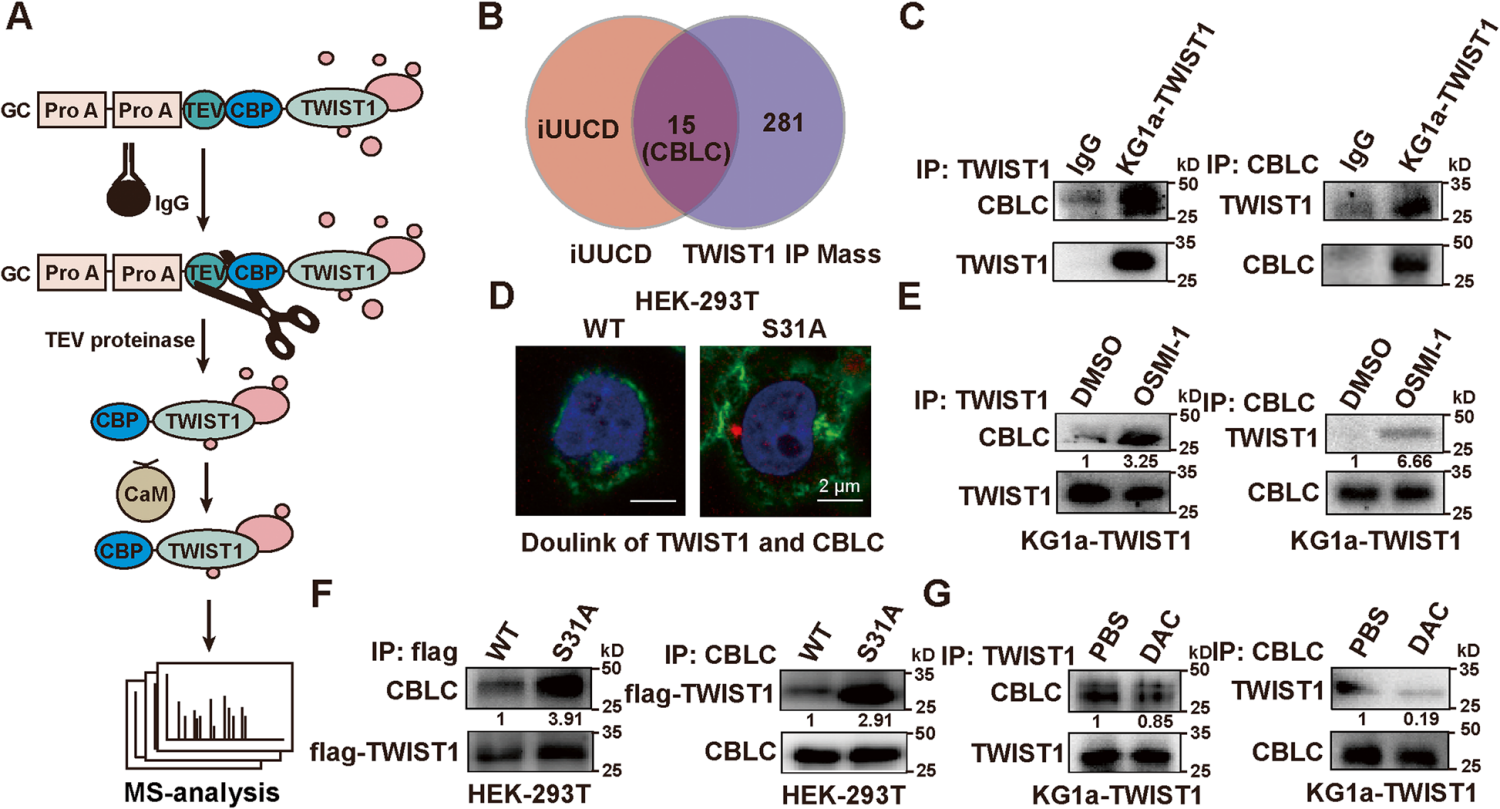
Inhibition of O-GlcNAcylation on TWIST1 binding to CBLC. **A** The identified proteins related to ubiquitination analyzed by integrated annotations for Ubiquitin and Ubiquitin-like Conjugation Database (iUUCD, http://iuucd.biocuckoo.org/). **B** The binding of CBLC and TWIST1 detected by Co-IP. Normal mouse IgG was used as a negative control. **C** Duo-link proximity ligation assay for interaction between CBLC and TWIST1 in HEK-293 T-WT and HEK-293 T-S31A. Red spot represents for the interaction. Nuclei was stained with DAPI (blue). Cytoskeleton was stained with phalloidine (green). Scale bar = 2 μm. **D** Western blotting analysis of interaction between CBLC and TWIST1 in KG1a-TWIST1 cells under 10 μg/mL OSMI-1 treatment for 48 h. **E** Western blotting analysis of interaction between CBLC and TWIST1 in HEK-293 T-WT and HEK-293 T-TWIST1-S31A cells. **F** Western blotting analysis of interaction between CBLC and TWIST1 in KG1a-TWIST1 under 20 ng/mL DAC treatment for 96 h

In our previous study, we identified that TWIST1 could interact with 281 proteins in KG1a-TWIST1 cells [6]. From integrated annotations for Ubiquitin and Ubiquitin-like Conjugation Database (iUUCD), we found 15 of these proteins were associated with ubiquitin–proteasome system (Fig. 4A, Table S3). Notably, TWIST1 interacted with Casitas B-Lineage Lymphoma Proto-Oncogene C (CBLC), an E3 ubiquitin ligase capable of transferring activated ubiquitin chains to specific lysine residues on substrates (Fig. 4A). Direct interaction of TWIST1 and CBLC was confirmed in KG1a-TWIST1 cells (Fig. 4B). With OSMI-1 treatment or mutation at S31, decreased O-GlcNAcylation promoted TWIST1 binding to CBLC, detected by IP/Western blotting and Doulink proximity ligation assay (Fig. 4C-E). In contrast, DAC treatment decreased the binding of TWIST1 and CBLC (Fig. 4F), suggesting that O-GlcNAcylation could stabilize TWIST1 by inhibiting its binding with CBLC.

### Effect of TWIST1 on transcription activation of OGT

**Fig. 5:**
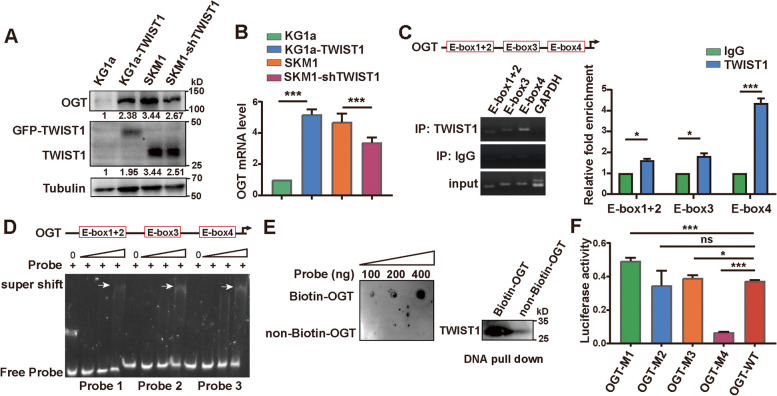
TWIST1 activates OGT transcription. **A**, **B** Expression of OGT at protein (**A**) and mRNA (**B**) levels in KG1a, KG1a-TWIST1, SKM1 and SKM1-shTWIST1 cells. **C** ChIP analysis for TWIST1 binding to E-box motifs of OGT promoter (0–2000 bp). GAPDH was used as negative control. **D** Validation of TWIST1 binding to OGT promoter by EMSA. **E** DNA pull down assay. Biotinylated OGT promoter region was incubated with cell nucleus lysate, enriched by magnetic beads conjugated with streptavidin and analyzed by western blotting. **F** Putative TWIST1 binding sites on wild-type (WT) and mutant sequences of 4 E-box (OGT-M1, OGT-M2, OGT-M3 and OGT-M4) of OGT promoter by dual luciferase reporter assay. *p < 0.05, ***p < 0.001, ns: not significant

Aforementioned, mRNA level of OGT was enhanced in CD34^+^ cells from MDS/AML patients who were non-responsive to DAC (Fig. 1C). We also found OGT expression was upregulated in TWIST1 overexpressed KG1a cells, but decreased in TWIST1 silenced SKM1 cells, at both protein (Fig. 5A) and mRNA level (Fig. 5B). TWIST1, as a basic helix-loop-helix (bHLH) TF, has been shown to bind the E-box elements of target genes. We speculated that TWIST1 may be involved the modulation of OGT expression at mRNA level. We found 4 E-box motifs (-CNNTTG-) at − 2.0 kb fragment of OGT promoter (Fig. 5C). ChIP assay and EMSA assay results showed the weak binding capacity of TWIST1 to No.1–3 E-box motifs, but strong binding to No.4 E-box motif of OGT promoter (Fig. 5C&D). It was confirmed that TWIST1 in the nuclear lysate could be pulled down by the biotinylated OGT promoter sequence (Fig. 5E), and OGT-M4 significantly decreased the binding capacity of TWIST1, compared to OGT-WT by luciferase reporter assay (Fig. 5F). These data indicate that TWIST1 promotes OGT transcription by binding to E-box at its promoter.

### The effect of O-GlcNAcylated TWIST1 on DAC resistance in vivo

**Fig. 6:**
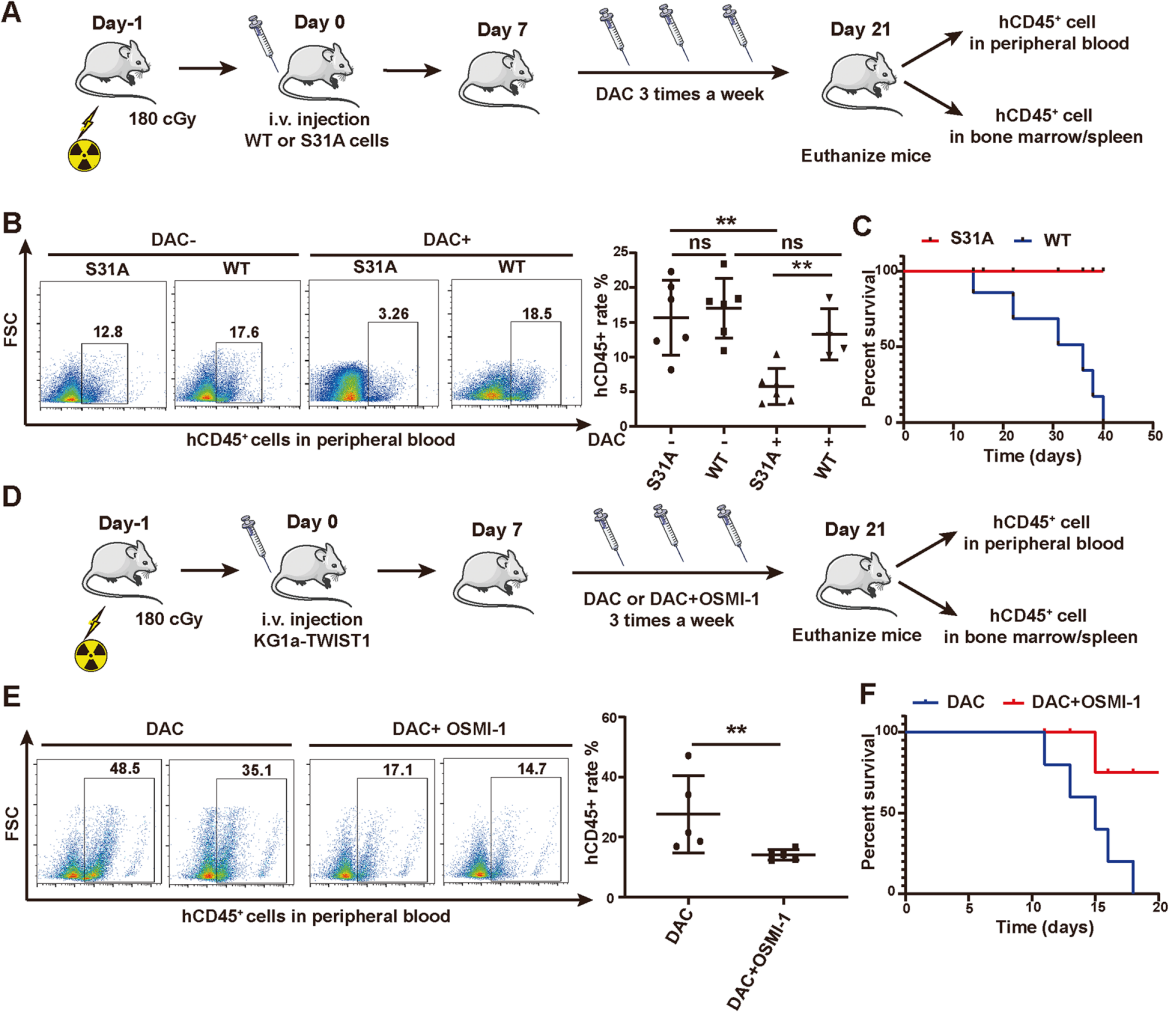
O-GlcNAcylation on TWIST1 affect the DAC resistance of MDS clone cells. **A** The scheme of the xenotransplantation mouse model. **B** FACS analysis of percentages of hCD45^+^ cells in peripheral blood. **C** Overall survival curve of mice injected KG1a-KO-TWIST1-WT or KG1a-KO-TWIST1-mutation. **D** The xenotransplantation mouse model. **E** FACS analysis for percentages of KG1a-TWIST1 cells in peripheral blood. **F** Overall survival curve of mice injected with KG1a cells and treated with DAC ± OSMI-1. **p < 0.01, ns: not significant

To verify whether O-GlcNAcylated TWIST1 could affect DAC resistance, S31A mutant and WT of flag-TWIST1 were overexpressed in TWIST1 knockout KG1a cells (KG1a-KO-TWIST1), respectively. The proliferation of S31A mutated cells were significantly inhibited when treated with DAC (Fig. S3A). In transplanted mouse model, injection of DAC also significantly decreased the proportion of hCD45^+^ cells in peripheral blood and improved the survival rate of mice injected with S31A mutated cells, compared to mice injected with WT cells (Fig. 6A-C). Consistently, decreased CD45^+^ cells were observed in bone marrows and spleens of mice injected with S31A mutated cells (Fig. S3B).

KG1a-TWIST1 was more resisted to DAC compared to KG1a, while decreased O-GlcNAcylation could eliminate the resistance to DAC of KG1a-TWIST1 (Fig. S3C). We then injected KG1a-TWIST1 into NSG mice and treated the mice with DAC and OSMI-1 (Fig. 6D). Co-injection of DAC and OSMI-1 significantly decreased the proportion of KG1a-TWIST1 cells in peripheral blood and improved the survival rate of mice (Fig. 6E, F). And less CD45^+^ cells were also observed in bone marrows and spleens of mice co-injected with DAC and OSMI-1 (Fig. S3D).

## Discussion

Drug resistance stands as a primary clinical hurdle in the effective management of MDS/AML patients, often arising during treatment with DAC, a prototypical DNMT inhibitor, consequently resulting in treatment failure [22, 23]. The characterization of specific molecular signatures can be used to better understand the drug response in MDS. In this study, we found an elevated O-GlcNAc and OGT expression in CD34^+^ cells of MDS/AML patients who were non-responsive to DAC.

O-GlcNAcylation, a ubiquitous form of PTMs, predominantly occurs on cytosol proteins and nucleoproteins. This process assumes a pivotal role in governing the configuration and functionality of numerous transcription factors, thereby constituting a crucial mechanism for orchestrating cellular processes [24–27]. Typically, O-GlcNAcylation levels in mammalian cells exhibit augmentation in reaction to environmental, physiological, or chemical stresses [28]. Accumulating evidence substantiates that hyper-O-GlcNAcylation significantly contributes to the emergence of chemotherapy resistance, as it oversees intracellular signaling and protein stability in diverse cancers such as ovarian cancer [29], colon cancer [30], liver cancer [31], pancreatic cancer [32] and breast cancer [33]. For example, escalated O-GlcNAcylation levels could be indicative of heightened resistance of cancer cells towards chemotherapy, whereas inhibition of O-GlcNAcylation reinstates chemotherapy susceptibility in breast cancer cells [33]. In this study, we found that suppression of OGT leads to an enhancement in chemotherapy responsiveness by downregulating TWIST1 expression.

TWIST1, as a potential oncogene, has been shown in previous studies to possess different PTMs including phosphorylation, acetylation and ubiquitination [34–38]. Through glycoproteomic analysis, we have discerned O-GlcNAcylation at Ser-31 on TWIST1. This O-GlcNAcylation event disrupts the interaction between TWIST1 and CBLC, thereby impeding degradation via the ubiquitin–proteasome system. Notably, these findings align with the notion that O-GlcNAcylation closely influences the stability of numerous proteins [39, 40]. As one core TF in epithelial-mesenchymal transition, TWIST1 binds to E-box sequences of target gene promoter and modulates their expression [41, 42]. Our investigation has unveiled the presence of four E-box regions within the OGT promoter, with the No.4 E-box motif being identified as the binding site for TWIST1. This interplay establishes a dual mechanism wherein TWIST1 activates OGT transcription, and reciprocally, OGT modifies TWIST1 at Ser-31 to bolster TWIST1's expression stability.

In line with our prior investigation, we observed elevated TWIST1 expression in CD34^+^ cells derived from MDS/AML patients who did not respond to DAC treatment. Furthermore, our previous research revealed TWIST1's ability to interact with DNMT3a, thereby exerting regulatory control over DAC resistance within MDS clone cells [6]. Here, our present findings indicate that a reduction in O-GlcNAcylation effectively eradicates the resistance to DAC. Remarkably, the combined administration of DAC and the OGT inhibitor OSMI-1 yields a notable reduction in the proportion of DAC-resistant cells in an in vivo model.

## Conclusions

**Fig. 7:**
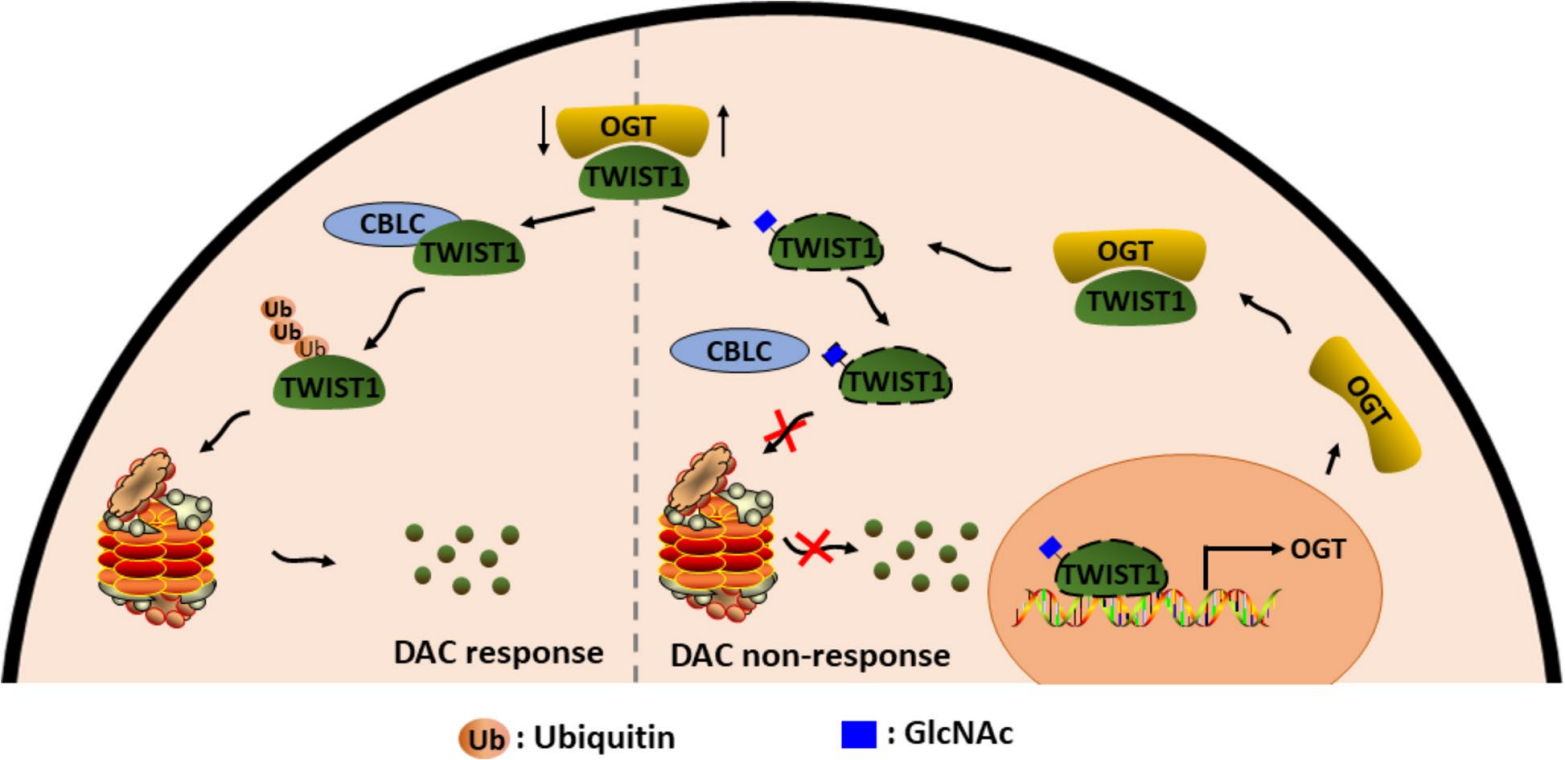
Schematic representation of reciprocal regulation of TWIST1 and OGT to mediate DAC resistance in MDS/AML

In conclusion, our study establishes a reciprocal regulatory relationship between TWIST1 and OGT that profoundly impacts decitabine efficacy in the context of MDS/AML (Fig. 7). Specifically, we have demonstrated that O-GlcNAcylation plays a pivotal role in stabilizing TWIST1 expression at the protein level, while TWIST1 exerts its influence on OGT by binding to its promoter and enhancing its transcriptional activity. This intricate interplay between TWIST1 and OGT emerges as a critical determinant of decitabine response.

### Supplementary Information

**Additional file 1: Fig. S1.** (A) OGT and O-GlcNAc in KG1a and DAC-resistant KG1a (KG1a-DAC-R). (B-C) OGT and O-GlcNAc in KG1a cells treated with DAC in dose and time dependent manners. (D-E) Expression of OGT and O-GlcNAc in SKM1 cells treated with DAC in dose and time dependent manners. (F-G) Cell apoptosis assay. CD34^+^ of MDS NR (F) and KG1a-DAC-R (G) cells were treated with DAC (20 ng/mL) and OSMI-1 (10 μg/mL) or PUGNAc (100 μM) for 48 h. **Fig. S2.** (A) TWIST1 expression and O-GlcNAc level in KG1a, KG1a-TWIST1 and KG1a-DAC-R. (B-C) TWIST1 expression in SKM1 cells treated with different concentrations of 10 μg/mL OSMI-1 (B) or 100 μM PUGNAc (C) for 48 h. (D-E) TWIST1 level in SKM1 cells treated with 10 μg/mL OSMI-1 (D) or 100 μM PUGNAc (E) at indicated times. (F-G) TWIST1 expression at mRNA level in KG1a-TWIST1 and SKM1 cell under 10 μg/mL OSMI-1 (F) or 100 μM PUGNAc (G) treatment for 48 h. (H-I) TWIST1 level in SKM1 after 10 μg/mL OSMI-1 (H) or 100 μM PUGNAc (I) treatment for 48 h and 1 μM CHX treatment for indicated times. (J-K) Ubiquitination of TWIST1 in KG1a-TWIST1 and SKM1 cells under 10 μg/mL OSMI-1 (J) or 100 μM PUGNAc (K) treatment. (L) TWIST1 level in KG1a-TWIST1 and SKM1 cell treated with 10 μg/mL OSMI-1, 1 μM CHX and 5 μM MG132 or 0.5 μM Baf-1. (M) TWIST1 level in KG1a-TWIST1 and SKM1 cell treated with 100 μM PUGNAc, 1 μM CHX and 5 μM MG132 or 0.5 μM Baf-1. **Fig. S3.** (A) Cell proliferation of KG1a-KO-TWIST1-WT or KG1a-KO-TWIST1-S31A under 20 ng/mL DAC treatment for 96 h. (B) CD45 level in the spleen and bone marrow tissues of mice injected KG1a-KO-TWIST1-WT or KG1a-KO-TWIST1-S31A cells detected by IHC staining. Scala bar = 100 μm. (C) Cell proliferation of KG1a and KG1a-TWIST1 under 20 ng/mL DAC and 10 μM OSMI-1 treatment for 96 h. (D) CD45 level in the spleen and bone marrow tissues of mice injected KG1a-TWIST1 cells and DAC ± OSMI-1 treatment detected by IHC staining. Scala bar = 100 μm. **Table S1.** Patients list. **Table S2.** Primer list. **Table S3.** Protein list.

**Additional file 2.**

**Additional file 3.**

## Data Availability

All data are contained within this article. Reagents and plasmids described in this article are available upon request.

## Abbreviations

MDS: Myelodysplastic syndromes
AML: Acute myeloid leukemia
DAC: Decitabine
TF: Transcription factors
OGT: O-GlcNAc transferase
OGA: Glycoside hydrolase O-GlcNAcase
HD: Healthy donors
DAC-NR: DAC non-response MDS/AML patients
DAC-R: DAC response MDS/AML patients
