# Supplementary material for: Reciprocal regulation of TWIST1 and OGT determines the decitabine efficacy in MDS/AML
Source: Cell Commun Signal. 2023 Sep 22;21:255. doi: 10.1186/s12964-023-01278-y (PMC10514931; doi:10.1186/s12964-023-01278-y)
Supplement: Supplementary file 2 — Additional file 1: Fig. S1. (A) OGT and O-GlcNAc in KG1a and DAC-resistant KG1a (KG1a-DAC-R). (B-C) OGT and O-GlcNAc in KG1a cells treated with DAC in dose and time dependent manners. (D-E) Expression of OGT and O-GlcNAc in SKM1 cells treated with DAC in dose and time dependent manners. (F-G) Cell apoptosis assay. CD34+ of MDS NR (F) and KG1a-DAC-R (G) cells were treated with DAC (20 ng/mL) and OSMI-1 (10 μg/mL) or PUGNAc (100 μM) for 48 h. Fig. S2. (A) TWIST1 expression and O-GlcNAc level in KG1a, KG1a-TWIST1 and KG1a-DAC-R. (B-C) TWIST1 expression in SKM1 cells treated with different concentrations of 10 μg/mL OSMI-1 (B) or 100 μM PUGNAc (C) for 48 h. (D-E) TWIST1 level in SKM1 cells treated with 10 μg/mL OSMI-1 (D) or 100 μM PUGNAc (E) at indicated times. (F-G) TWIST1 expression at mRNA level in KG1a-TWIST1 and SKM1 cell under 10 μg/mL OSMI-1 (F) or 100 μM PUGNAc (G) treatment for 48 h. (H-I) TWIST1 level in SKM1 after 10 μg/mL OSMI-1 (H) or 100 μM PUGNAc (I) treatment for 48 h and 1 μM CHX treatment for indicated times. (J-K) Ubiquitination of TWIST1 in KG1a-TWIST1 and SKM1 cells under 10 μg/mL OSMI-1 (J) or 100 μM PUGNAc (K) treatment. (L) TWIST1 level in KG1a-TWIST1 and SKM1 cell treated with 10 μg/mL OSMI-1, 1 μM CHX and 5 μM MG132 or 0.5 μM Baf-1. (M) TWIST1 level in KG1a-TWIST1 and SKM1 cell treated with 100 μM PUGNAc, 1 μM CHX and 5 μM MG132 or 0.5 μM Baf-1. Fig. S3. (A) Cell proliferation of KG1a-KO-TWIST1-WT or KG1a-KO-TWIST1-S31A under 20 ng/mL DAC treatment for 96 h. (B) CD45 level in the spleen and bone marrow tissues of mice injected KG1a-KO-TWIST1-WT or KG1a-KO-TWIST1-S31A cells detected by IHC staining. Scala bar = 100 μm. (C) Cell proliferation of KG1a and KG1a-TWIST1 under 20 ng/mL DAC and 10 μM OSMI-1 treatment for 96 h. (D) CD45 level in the spleen and bone marrow tissues of mice injected KG1a-TWIST1 cells and DAC ± OSMI-1 treatment detected by IHC staining. Scala bar = 100 μm. Table S1. Patients list. Table S2. Primer list. Table S3. Protein l [file 12964_2023_1278_MOESM1_ESM.docx]

**Supplementary information**

**Title: Reciprocal regulation of TWIST1 and OGT determines the decitabine efficacy in MDS/AML**

Hongjiao Li^1#^, Yi Wang^2#^, Shuang Feng^1^, Kaijing Chang^1^, Xinwen Yu^1^, Fenfang Yang^1^, Haozhe Huang^1^, Yuanbo Wang^1^, Xiang Li^3*^, Feng Guan^1*^

1. Key Laboratory of Resource Biology and Biotechnology Western China, Ministry of Education; Provincial Key Laboratory of Biotechnology, College of Life Sciences, Northwest University, Xi'an, 710069,China.

2. Department of Hematology, Provincial People’s Hospital, Xi’an, 710068, China.

3. Institute of Hematology, School of Medicine, Northwest University, Xi’an, 710069,China

# These authors contributed equally to this study.

Correspondence to Xiang Li (e-mail: xiangli@nwu.edu.cn) or Feng Guan (e-mail: guanfeng@nwu.edu.cn), Tel: +86-29-88303534, College of Life Science, Northwest University, 229 Taibai North Road, Xi’an, Shaanxi 710069, China

**Supplementary Figures**


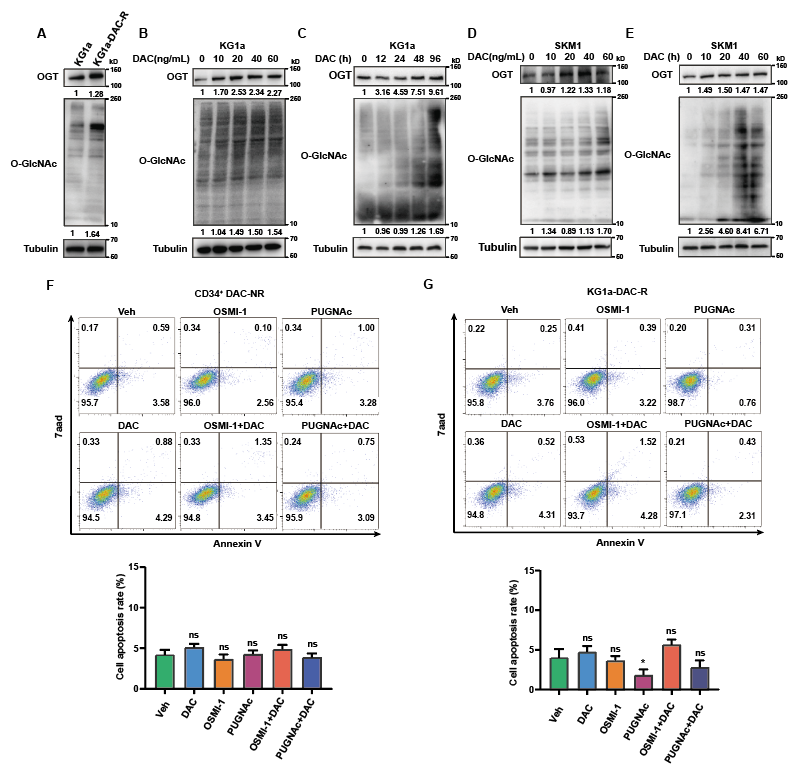


**Fig. S1 (A)** OGT and O-GlcNAc in KG1a and DAC-resistant KG1a (KG1a-DAC-R). **(B-C)** OGT and O-GlcNAc in KG1a cells treated with DAC in dose and time dependent manners. **(D-E)** Expression of OGT and O-GlcNAc in SKM1 cells treated with DAC in dose and time dependent manners. **(F-G)** Cell apoptosis assay. CD34^+^ of MDS NR (F) and KG1a-DAC-R (G) cells were treated with DAC (20 ng/mL) and OSMI-1 (10 μg/mL) or PUGNAc (100 μM) for 48 h.


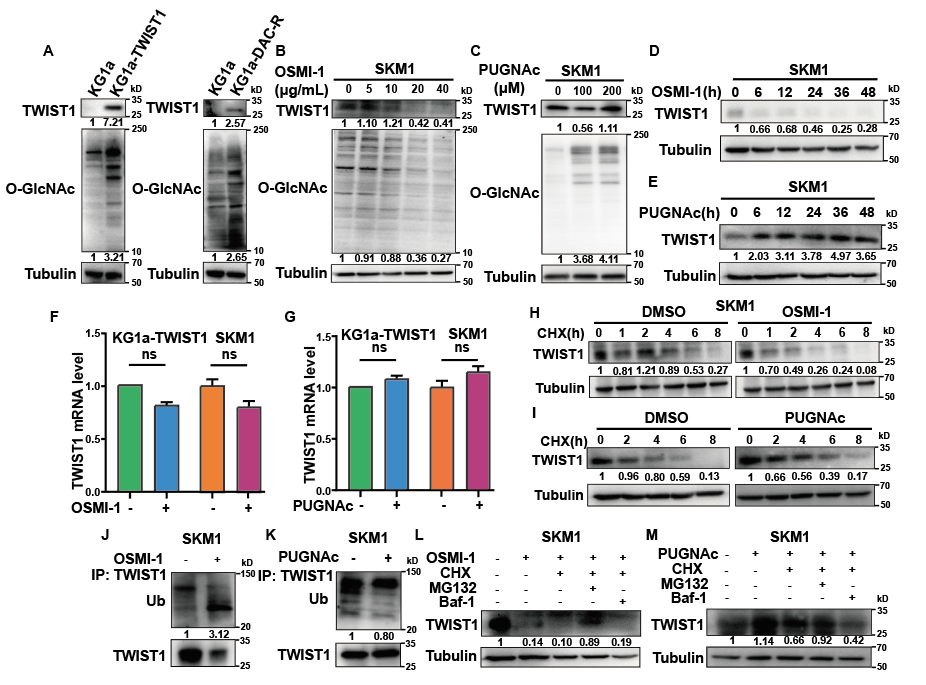


**Fig. S2** **(A)** TWIST1 expression and O-GlcNAc level in KG1a, KG1a-TWIST1 and KG1a-DAC-R. **(B-C)** TWIST1 expression in SKM1 cells treated with different concentrations of 10 μg/mL OSMI-1 (B) or 100 μM PUGNAc (C) for 48 h. **(D-E)** TWIST1 level in SKM1 cells treated with 10 μg/mL OSMI-1 (D) or 100 μM PUGNAc (E) at indicated times. **(F-G)** TWIST1 expression at mRNA level in KG1a-TWIST1 and SKM1 cell under 10 μg/mL OSMI-1 (F) or 100 μM PUGNAc (G) treatment for 48 h. **(H-I)** TWIST1 level in SKM1 after 10 μg/mL OSMI-1 (H) or 100 μM PUGNAc (I) treatment for 48 h and 1 μM CHX treatment for indicated times. **(J-K)** Ubiquitination of TWIST1 in KG1a-TWIST1 and SKM1 cells under 10 μg/mL OSMI-1 (J) or 100 μM PUGNAc (K) treatment. **(L)** TWIST1 level in KG1a-TWIST1 and SKM1 cell treated with 10 μg/mL OSMI-1, 1 μM CHX and 5 μM MG132 or 0.5 μM Baf-1. **(M)** TWIST1 level in KG1a-TWIST1 and SKM1 cell treated with 100 μM PUGNAc, 1 μM CHX and 5 μM MG132 or 0.5 μM Baf-1.
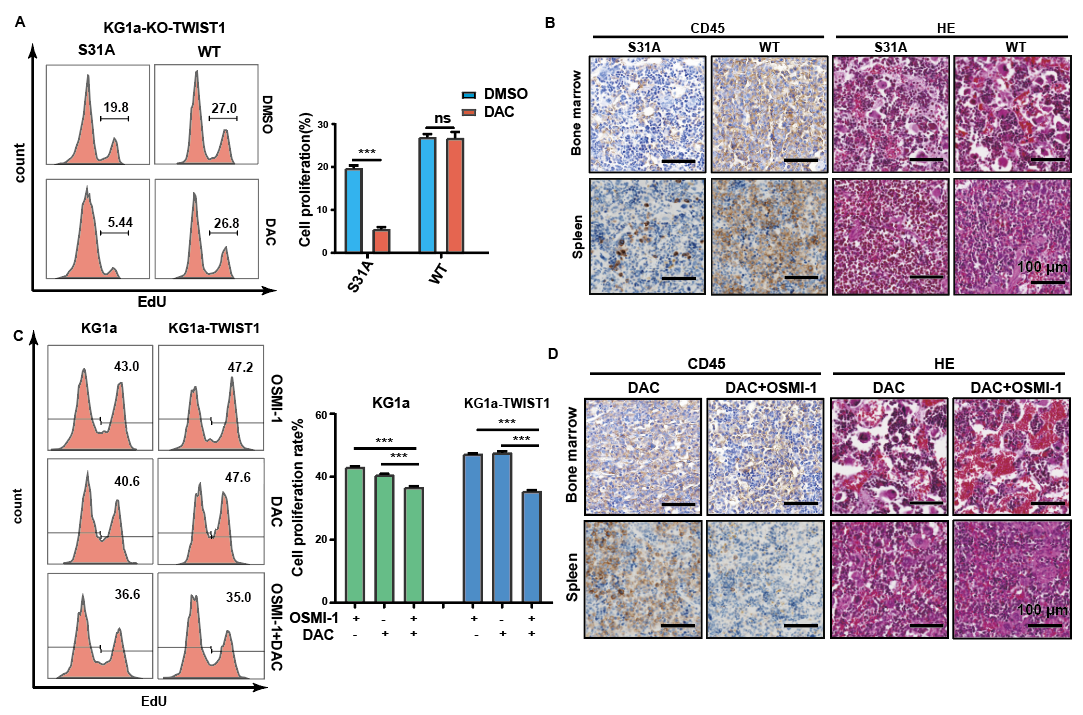
 **Fig. S3 (A)** Cell proliferation of KG1a-KO-TWIST1-WT or KG1a-KO-TWIST1-S31A under 20 ng/mL DAC treatment for 96 h. **(B)** CD45 level in the spleen and bone marrow tissues of mice injected KG1a-KO-TWIST1-WT or KG1a-KO-TWIST1-S31A cells detected by IHC staining. Scala bar = 100 μm. **(C)** Cell proliferation of KG1a and KG1a-TWIST1 under 20 ng/mL DAC and 10 μM OSMI-1 treatment for 96 h. **(D)** CD45 level in the spleen and bone marrow tissues of mice injected KG1a-TWIST1 cells and DAC ± OSMI-1 treatment detected by IHC staining. Scala bar = 100 μm.

**Table S1 Patients list**

| **Diagnosis** | **Age (yr)** | **Gender** | **Cytogenetics** | **BM cellularity** | **Marrow blast count** |
| --- | --- | --- | --- | --- | --- |
| AML | 50 | F | t(8, 21) | hyper | 63.5% |
| AML | 77 | F | normal | hype | 24% |
| AML | 64 | M | normal | normal | 35% |
| AML | 73 | F | normal | hyper | 43% |
| AML | 48 | M | normal | hyper | 78% |
| AML | 48 | M | normal | hyper | 45% |
| AML | 64 | M | normal | hyper | 91% |
| AML | 31 | F | normal | hyper | 53% |
| AML | 48 | F | normal | hyper | 61% |
| AML | 53 | F | normal | hype | 27% |
| AML | 62 | F | t(8, 21) | normal | 30% |
| AML | 48 | M | t(8, 21) | hyper | 23.5% |
| AML | 58 | M | normal | hyper | 29% |
| AML from MDS | 78 | F | normal | normal | 40% |
| AML from MDS | 80 | M | t(8, 21) | hyper | 36% |
| MDS | 54 | M | normal | normal | 16.8% |
| MDS | 35 | M | normal | hyper | 12% |
| MDS | 31 | M | normal | hyper | 14% |
| MDS | 56 | M | normal | hype | 13% |
| MDS | 58 | M | +8 | normal | 6.5% |
| MDS | 66 | F | complex | normal | 23% |
| MDS | 72 | F | normal | normal | 17% |
| MDS | 65 | M | normal | hype | 15% |
| MDS | 65 | M | normal | normal | 6.5% |
| MDS | 62 | M | normal | normal | 8% |

**Table S2 Primer list**

| Primers | Sequences |
| --- | --- |
| OGT-Qpcr-F | TGCTTGGACACTCCACTCTG |
| OGT-Qpcr-R | GAGCCGCTCTAGTTCCATTG |
| TWIST1-XhoI-F | CCGCTCGAGCGGATGATGCAGGACGTGTCCA |
| TWIST1-BamHI-R | GCGCCTAGGCGCCTAGTGGGACGCGGACATG |
| OGT-Ebox-F1 | CGTAAGAACCGATTTGTTCAAGGAAG |
| OGT-Ebox-R1 | CACATTGTCACAGTCGGCGAT |
| OGT-Ebox-F2 | ATGGCTGCCTTCGGAGTG |
| OGT-Ebox-R2 | CACCTTCAATGTTGGCCGTATAGC |
| OGT-Ebox-F3 | CGATTTTGAAATTTCAGGCTATACGGC |
| OGT-Ebox-R3 | GGCCATCGATTTCAGCACTTCGA |
| OGT-M1-A-F | GTTTAGGACGATGGCAAGTGAATTCATTTATC |
| OGT-M1-A-R | GAATTCACTTGCCATCGTCCTAAACCACA |
| OGT-M1-B-F | GTTTAGGCAGATGGACAGTGAATTCATTTATC |
| OGT-M1-B-R | GAATTCACTGTCCATCTGCCTAAACCACA |
| OGT-WT-F | CGTGCTAGCCCGGGCTCGAGGTAATACTTTCTTGCTGATGTGGCAAGC |
| OGT-WT-R | AGTACCGGAATGCCAAGCTTATCTGCCAAGGTAGAACGGGACA |
| OGT-M2-F | AGTTATTAAAACTTTGCCCTATCACCGTG |
| OGT-M2-R | GTGATAGGGCAAAGTTTTAATAACTCAGGG |
| OGT-M3-F | TCTGCATTACACTGGCCCTGTGAAA |
| OGT-M3-R | GCCAGTGTAATGCAGACACCTTCA |
| OGT-promoter-F | GTGATACTTTGTTACACAGCAG |
| OGT-promoter-R | TACAGGCTTCAAACCCTG |

**Table S3 Protein list**

| Protein name | Function |
| --- | --- |
| GRWD1 | E3 adaptor/Cullin RING/DCX/DWD |
| C4A | E3 adaptor/Cullin RING/Other |
| CBLC | E3 activity/RING/RING |
| CUL4A | E3 adaptor/Cullin RING/Cullin |
| CUL4B | E3 adaptor/Cullin RING/Cullin |
| DDB1 | E3 adaptor/Cullin RING/DCX/DDB1 |
| DDX5 | UBD/CARD |
| RPS27A | ULD/UBL/NEDD8 |
| HLTF | E3 activity/RING/RING |
| MLK4 | UBD/SH3 |
| MLL | E3 activity/RING/PHD |
| PHIP | E3 adaptor/Cullin RING/DCX/DWD |
| RBBP4 | E3 adaptor/Cullin RING/DCX/DWD |
| WBP7 | E3 activity/RING/PHD |
| PARP1 | DUB/Other |
