# Supplementary figures and images for: Reciprocal regulation of TWIST1 and OGT determines the decitabine efficacy in MDS/AML
Source: Cell Commun Signal. 2023 Sep 22;21:255. doi: 10.1186/s12964-023-01278-y (PMC10514931; doi:10.1186/s12964-023-01278-y)

Figure 2

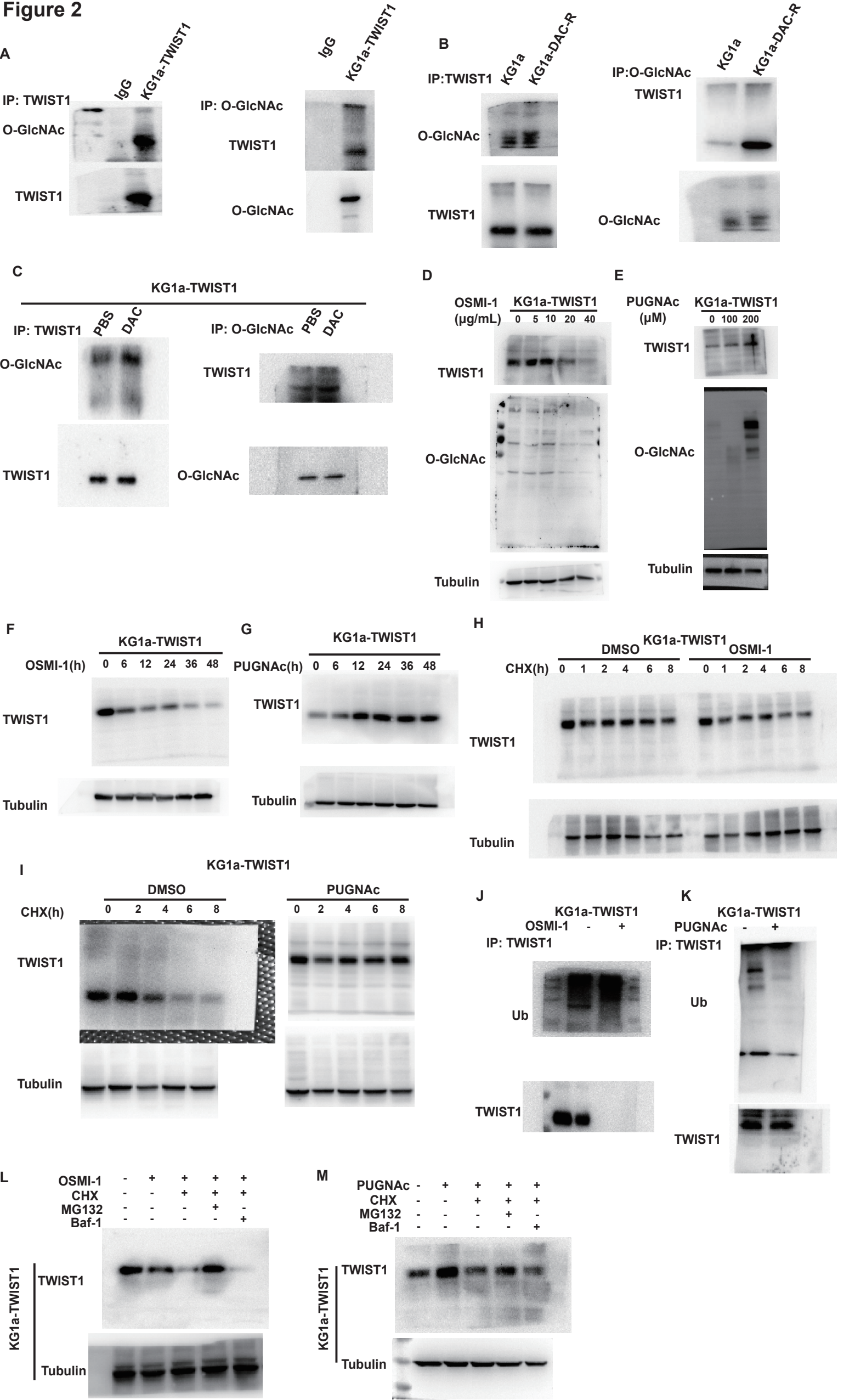

Figure 3

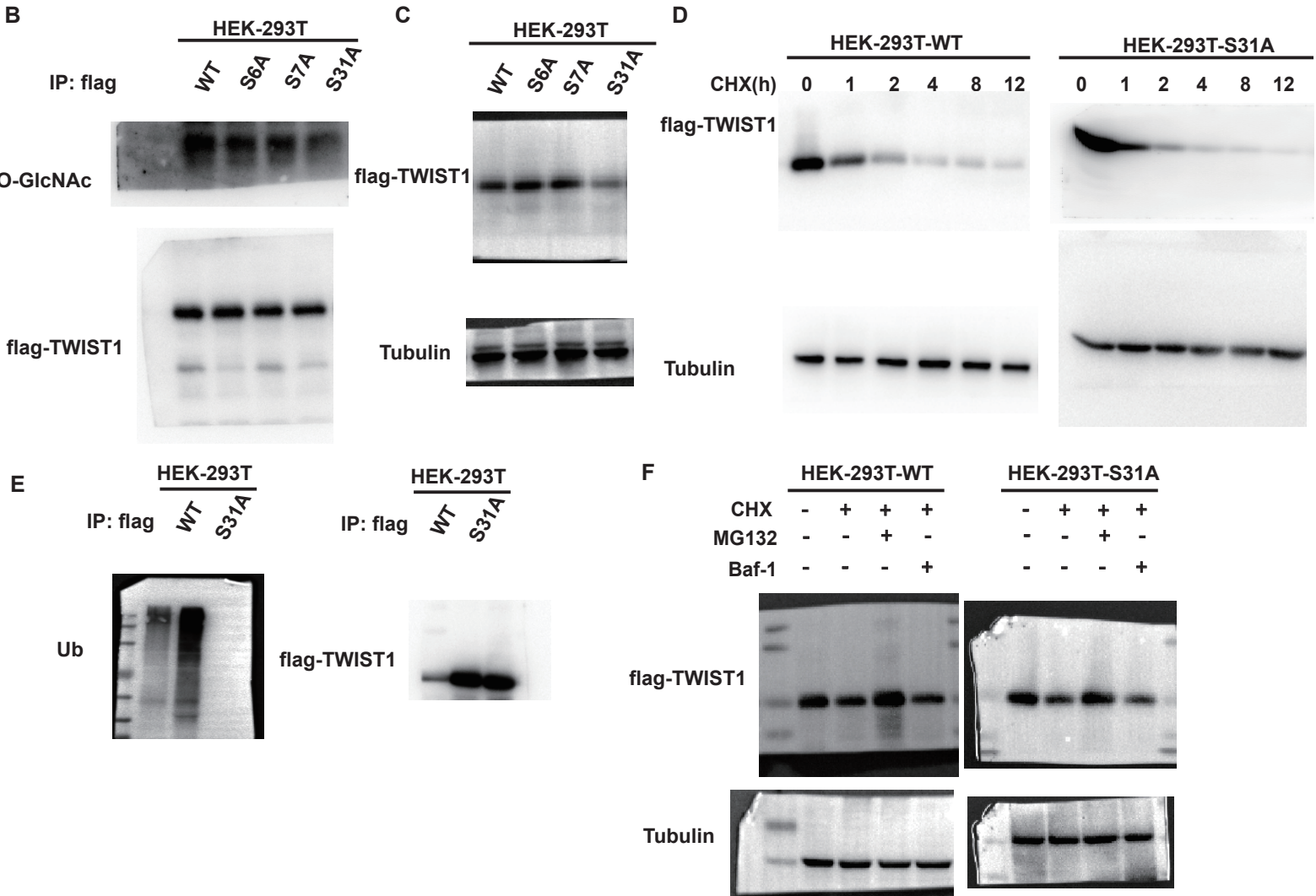

Figure 4

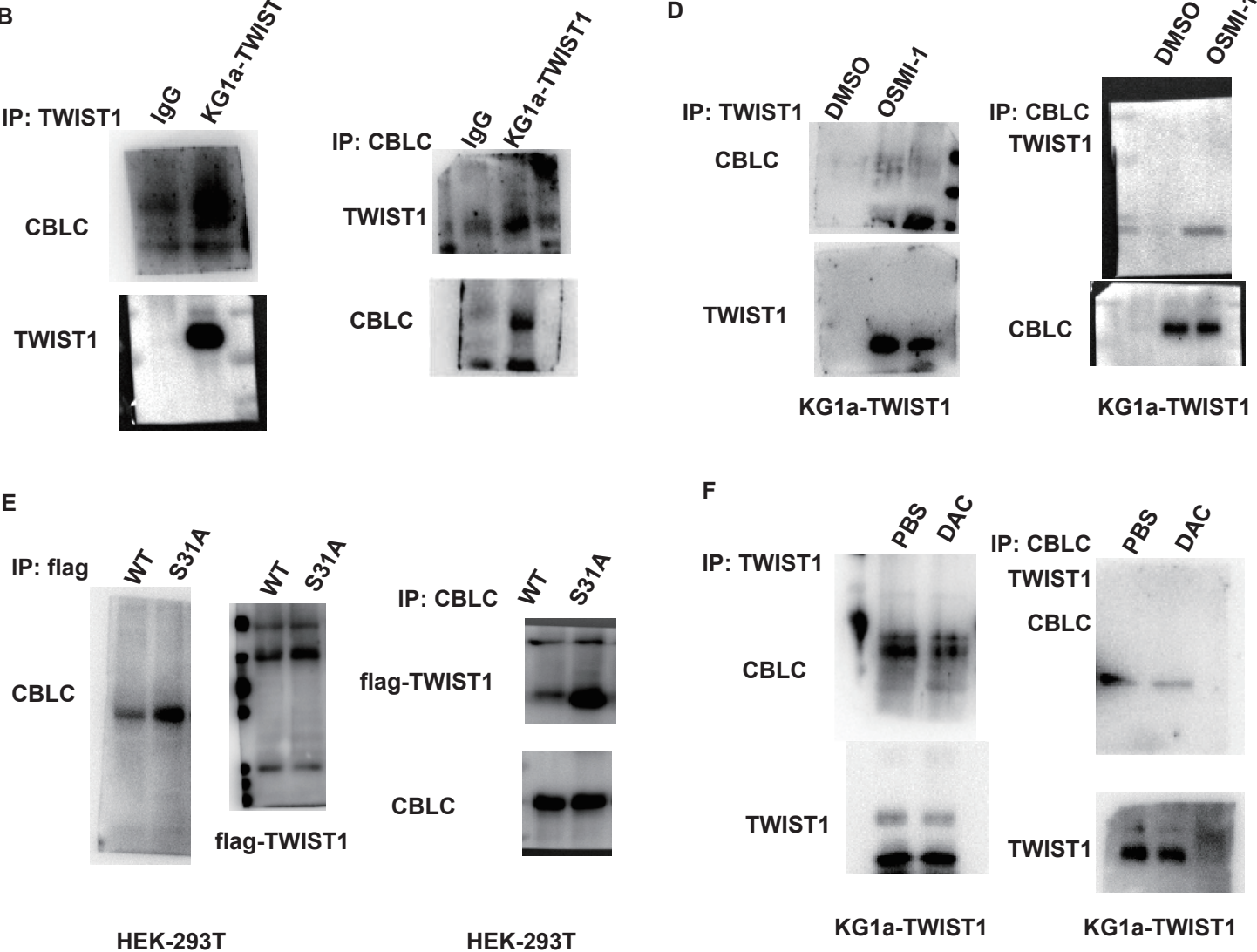

Figure 5

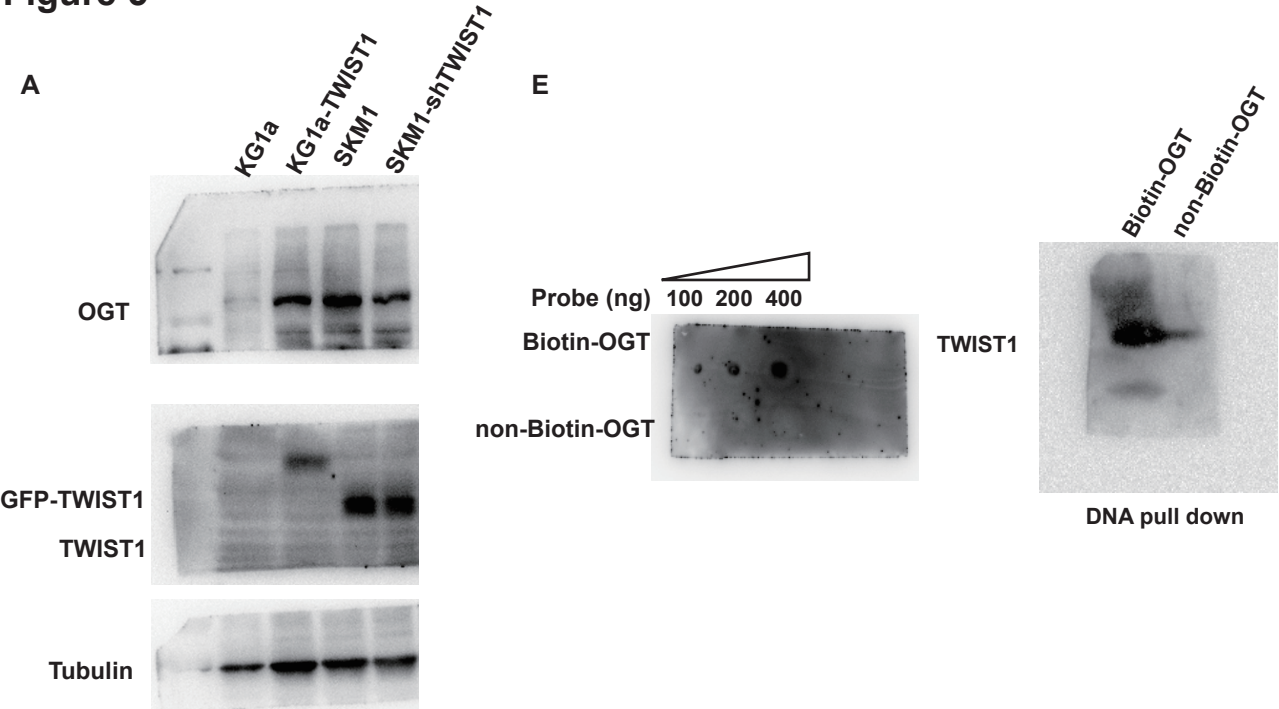

Supplement: Supplementary file 3 — Additional file 2. [file 12964_2023_1278_MOESM2_ESM.pdf]

Figure S1

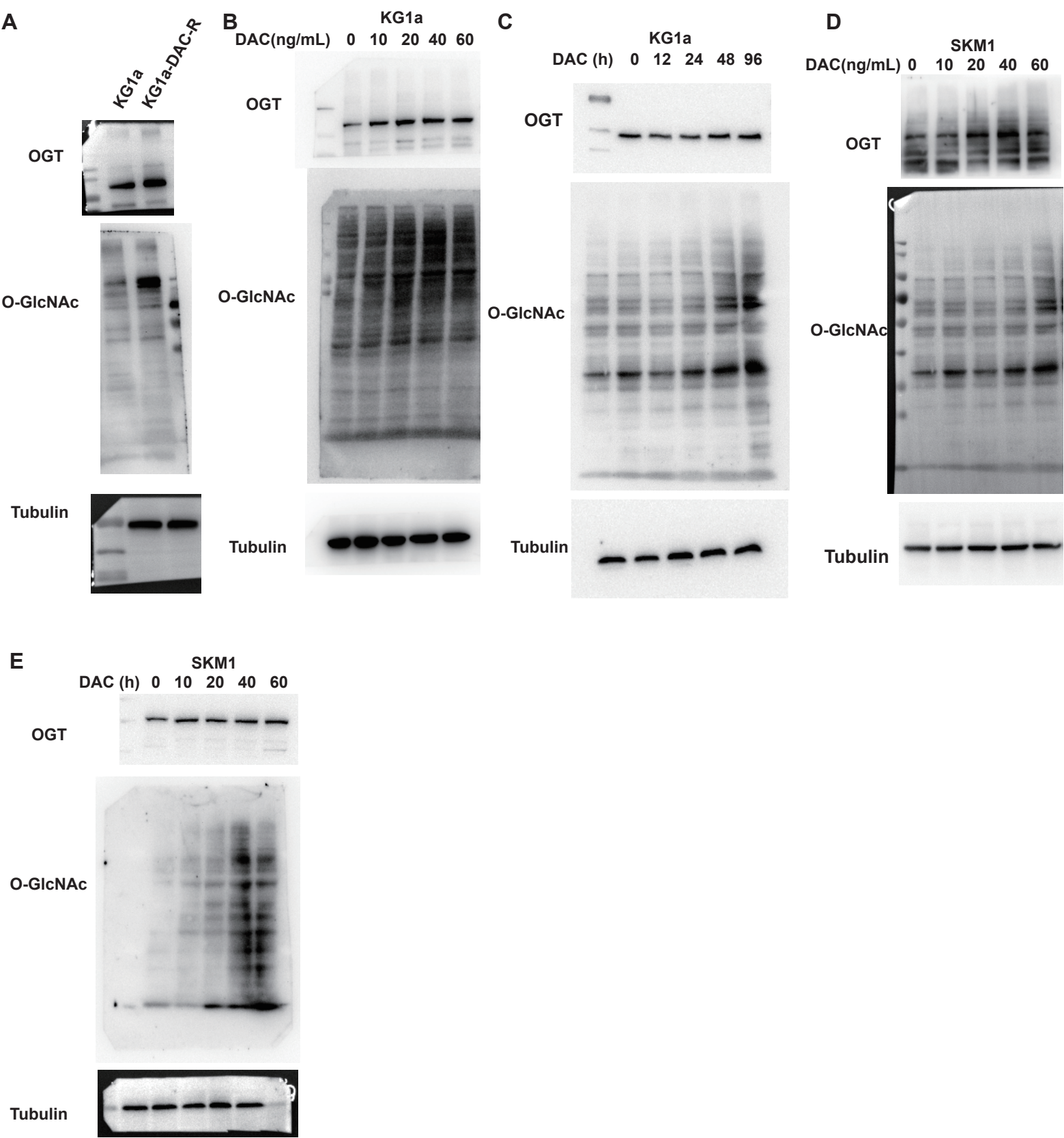

Figure S2

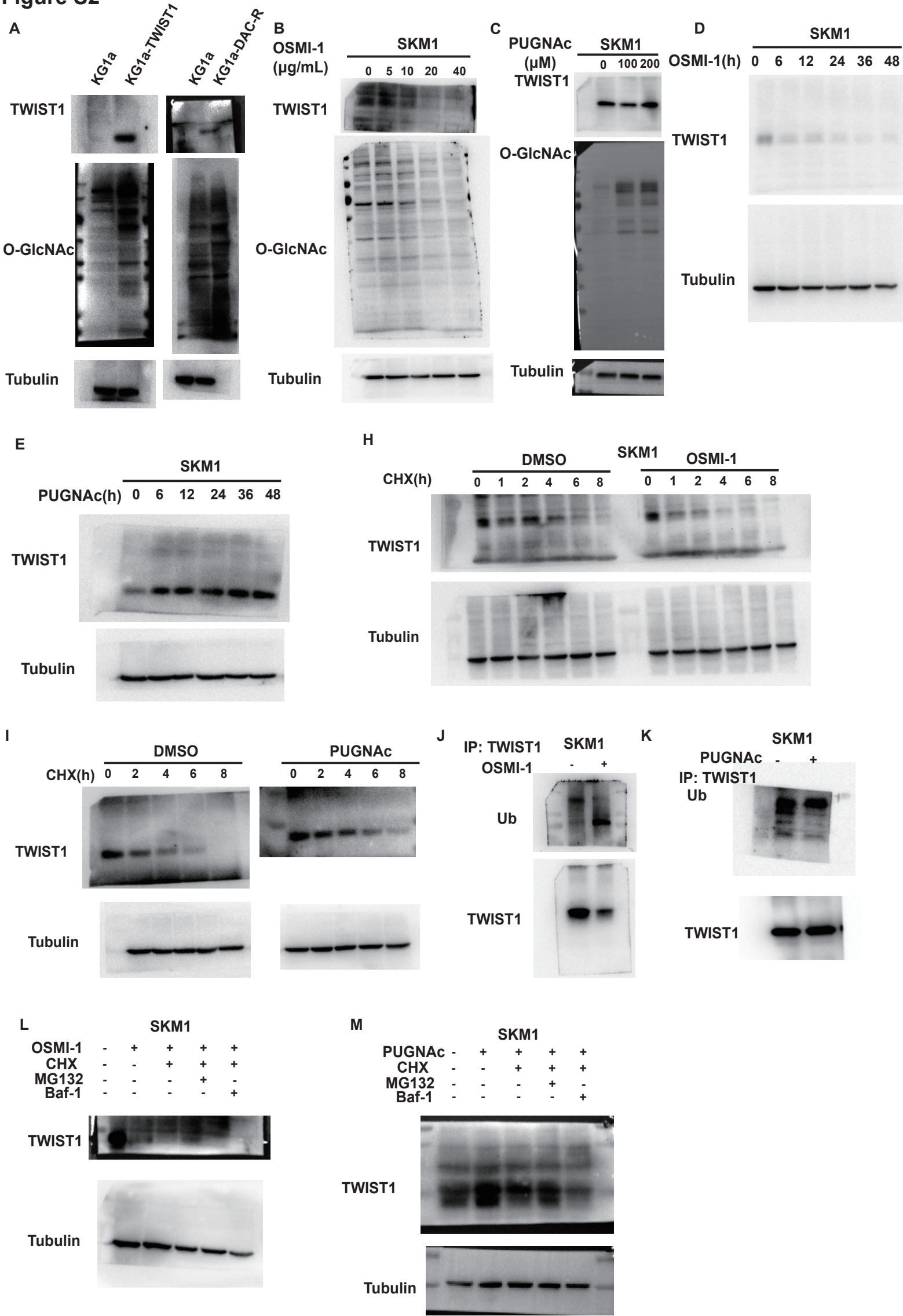

Supplement: Supplementary file 4 — Additional file 3. [file 12964_2023_1278_MOESM3_ESM.pdf]
